# The Presence of Pilonidal Sinus Infection Before Surgery Versus Other Factors as Predictors of Sinus Recurrence After Surgical Excision: A Retrospective Study

**DOI:** 10.7759/cureus.77080

**Published:** 2025-01-07

**Authors:** Andro A Awed, Mostafa B Duhmaj, Hamed Al-Aamri, Mohamed Elawdy, Mazin A Arif, Farid A Meanji, Aida S Al Jamoudi, Samiya Al Hattali, Khalid S Al Aamri

**Affiliations:** 1 General Surgery, Nizwa Hospital/Ministry of Health, Nizwa, OMN; 2 Surgery, Nizwa Hospital/Ministry of Health, Nizwa, OMN; 3 General Surgery, Ibra Hospital, Ibra, OMN; 4 Urology, Sohar Hospital/Ministry of Health, Sohar, OMN

**Keywords:** infection, pilonidal sinus, recurrence, sacrococcygeal, surgery

## Abstract

Aims and objectives

Pilonidal sinus is a frustrating condition because of its high recurrence rate. In our study, we aimed to find a correlation between the different variables during surgical management of pilonidal sinus with its recurrence within a three-year follow-up period.

Methods

A retrospective analysis was conducted on the hospital’s electronic data retrieving 100 patients who were admitted with sacrococcygeal pilonidal sinus in three years and underwent surgical excision.

Results

Their mean age was 23.8 years, and 81 patients were males. They underwent four different surgical techniques, 26 of them had one or more signs of sinus infection, and we observed a 20% recurrence of the disease within the follow-up period. Bivariate analysis revealed no correlation between the recurrence of the sinus with either the type of surgery performed the preoperative antibiotics course, or the drain insertion after excision. There is a statistically significant relationship between any signs of infection prior to surgery and the recurrence regardless of the surgical technique (p = 0.001).

Conclusion

Signs of sinus infection before surgery are statistically significant predictors of the recurrence of the disease. On the other hand, there is no correlation between the type of surgical technique, the preoperative course of antibiotics, and the insertion of a drain after surgery with the recurrence.

## Introduction

The pilonidal sinus of the sacrococcygeal region is a common, chronic disease and has an incidence of 26 per 100,000 people with more prevalence in males than females (2.2-4 times) [1]. It affects young adults at working age more frequently [2]. When there is no infection, the sinus is asymptomatic, but when the sinus becomes infected, it shows symptoms ranging from intermittent pain and tenderness to serous-purulent discharge and may result in forming an abscess [3]. Because this tract is characterized by repeated attacks of infection and frequent inflammation, ranging from acute to chronic, it has many complications including chronic non-healing wounds, recurrent abscesses, and even squamous cell carcinoma [4]. Although many treatment options have been used to eradicate this disease, pilonidal sinus treatment remains debatable, and there is no current widely accepted method that is known to decrease the complications and the recurrence rates [5]. The majority of the previous studies have focused on the correlation between the different surgical techniques and the recurrence rate. Although variable recurrence rates have been reported for pilonidal sinus with different ranges with some evidence suggesting that recurrence is associated with the surgical technique and correlated with the length of follow-up as well, these data remain in conflict which brings up the question about the validity of the reported recurrence rates associated with different surgical procedures [6].

In our study, we aimed to determine the significance of the signs of sinus infection before surgery, the type of surgical technique, the preoperative course of antibiotics, and the use of a drain after sinus excision as factors in the management process that could predict the recurrence of the pilonidal sinus after surgical excision.

## Materials and methods

Methods

This study was conducted at Nizwa Hospital, which is a 555 bedded tertiary care center, under the Ministry of Health, Sultanate of Oman. After obtaining an Ethical approval (Research and Ethical Review and Approval Committee, Al Dakhliya Governorate, Ministry of Health, Sultanate of Oman dated December 23, 2024: approval number-DGHS/R&S/PROPOSAL-APPROVED/23.12.2024), a retrospective analysis was conducted using the hospital’s electronic database retrieving the data of 129 patients who were admitted with pilonidal sinus in the sacrococcygeal region and underwent elective surgical excision between January 2015 and December 2017. All of them were admitted a day before surgery and underwent surgical excision of the sinus. These patients were followed up three years post-surgery and were either contacted or seen and examined in our outpatient clinics to determine whether the sinus was recurrent.

Inclusion and exclusion criteria

The following categories of patients were included in the study: those who did not receive antibiotics before hospital admission, all patients who received a five- to seven-day full course of oral antibiotics before hospital admission, and all patients who received a five- to seven-day full course of oral antibiotics after surgery. However, patients who received antibiotics before hospital admission less than five days as an incomplete course of antibiotics to treat any existing infection, patients who did not receive a full course of antibiotics for five to seven days after hospital discharge, and all patients who could not be contacted three years after hospital discharge were excluded from the study.

Preoperative evaluation

The antibiotic history was obtained with some patients having received a full course of oral antibiotics preoperatively. This was based on the practice of the surgeon who performed the operation and was either cefuroxime or amoxicillin/clavulanic acid. At the time of hospital admission (one day before surgery), a physical examination was performed including a local sinus examination in order to observe the presence of signs of infection at the sacrococcygeal region including purulent discharge, offensive odor, or a red and tender area. All patients had the basic laboratory tests including complete blood count, renal function tests, and urine analysis prior to surgery.

Operative details

All patients underwent surgery the next day after admission with either general or spinal anesthesia in a prone position with the buttocks laterally retracted with tape for better exposure. Within an hour before the skin incision, all the patients received a single dose of antibiotic intravenously. The same disinfection solution was used in skin preparation in all patients, which was 10% povidone-iodine. Methylene blue was injected into the sinuses of all patients to determine the extent of it. According to the decision of the operating surgeon, our patients underwent one of four different surgical techniques.

Complete Excision of the Sinus With Midline Primary Closure of the Wound

A symmetrical elliptical incision was made around the sinus at the midline including all the external pits of the sinus. The whole tissue surrounding the sinus to the presacral fascia was removed, and the wound was primarily closed. Patients were instructed to an alternative day wound dressing at the local health center and to have their wound stitches removed after two weeks of surgery.

Complete Excision of the Sinus Leaving the Wound Open to Be Closed by Secondary Intention

A symmetrical elliptical incision was made around the sinus including all the external pits of the sinus. The whole tissue surrounding the sinus to the presacral fascia was removed, and the wound was left open and packed to be closed by secondary intention. The first postoperative dressing was performed with normal saline and non-adhesive paraffin gauze dressing. Patients were informed to have daily dressing with the same material after hospital discharge at their local health center till complete wound healing.

Complete Excision of the Sinus With an Advancement Flap (Karydakis Procedure) and Primary Closure of the Wound

An asymmetric elliptical incision was made around the sinus including all the external pits of the sinus. Complete excision of the sinus up to the presacral fascia was done, and an advancement flap through the whole incision was created from one of the lateral wound edges with 1-2 cm depth. Then, absorbable sutures were used in the fatty tissue of the prepared flap to the other wound edge to obliterate the midline cleft. Then, the skin was closed with a non-absorbable suture. Patients were informed to have alternative day wound dressing at the local health center and to have their wound stitches removed after two weeks of surgery.

Complete Excision of the Sinus With Limberg Flap Creation and Closure of the Wound

A vertical rhomboidal incision was made around the sinus at the midline including all the external pits of the sinus. The whole tissue surrounding the sinus to the presacral fascia was removed, and an incision was made at one lateral angle to create a flap, which was used to close the defect. Then, the skin was closed with a non-absorbable monofilament suture. Patients were informed to have alternative day wound dressing at the local health center and to have their wound stitches removed after two weeks of surgery.

Some of the patients had a drain inserted into their wounds, and others had no drains. At the end of surgery, all the patients had their sinus sent for histopathological examination to confirm complete sinus excision.

Postoperative review

Patients remained admitted at least one day post-surgery in the hospital and were then discharged. They were prescribed a full course of oral antibiotics at the time of discharge. After hospital discharge, most of our patients had a follow-up visit in the outpatient clinic with the first appointment at 10-15 days post-operative and the subsequent visits were determined according to the patient’s clinical findings. They were then contacted three years post-surgery, either by phone or seen in our outpatient clinic, to determine if they had a recurrence of the disease. All the patients who were included in our study had their histopathology results after surgery which showed complete sinus excision.

Outcome

The primary outcome of the study was to review the potential predictors of the recurrence of the sinus after surgical excision. Thus, data were collected from the hospital's electronic system from patients’ files. The diagnosis of a recurrent sinus was made on the basis of the clinical features, such as fistula formation or development of an abscess and purulent discharge from the wound scar within three years post-surgery.

The statistical analysis was performed by using Statistical Package for Social Sciences (SPSS) 25 (IBM Corp., Armonk, NY). The data were expressed as mean +/- standard deviation (SD) or percentages, depending on the variable. The chi-square test was used for nominal data, and Mann-Whitney U statistical analysis was used for ordinal data and non-parametric numerical data. A p-value <0.05 was considered statistically significant.

## Results

Of the 129 patients, 17 patients received antibiotics before hospital admission in less than a five-day course, eight patients did not receive a course of antibiotics for five to seven days after hospital discharge, four patients missed follow-up, and 100 were successfully traced. The records of these 100 patients were eligible for review. We had a recurrence of the disease within three years post-surgery follow-up period at a rate of 20% with patients having either fistula formation developed an abscess or had purulent discharge from the wound scar. Out of the 100 patients who underwent surgical excision for sacrococcygeal pilonidal sinus in our study had a mean age of 23.8 ±6.5 years (mean ±SD), with the maximum age to present with pilonidal sinus was 42 years old, the majority of the patients with the disease were aged between 20 and 24 years old (39%).

Out of the 100 patients, 81 patients were male. There was no statistically significant difference between groups in terms of gender or age about the recurrence of the sinus after surgical excision (p>0.05). On hospital admission one day before surgery, 26 of the 100 patients showed one or more sinus infection signs. These patients had a high recurrence rate of the disease compared to those who did not show any signs of sinus infection, and there was a significant difference with p-value = 0.001. Out of the 100 patients, only 18 received a five to seven-day course of antibiotics before surgery. In terms of recurrence, there was no significant difference between them and patients who did not receive antibiotics (Table 1).

**Table 1:** Demographic and clinical characteristics of patients Values are presented as numbers (%) * Mann-Whitney U test, / chi-square test. p<0.05 is considered statistically significant.

Variables		Bivariate analysis				
		Recurrence of sinus				P-value*
		No, N:80(%)		Yes, N:20(%)		
Gender	Male (81)	67	(83)	14	(17)	0.140
	Female (19)	13	(68)	6	(32)	
Age (years)	14 (2)	2	(100)	0	(0)	0.681
	15-19 (26)	18	(69)	8	(31)	
	20-24 (39)	31	(79)	8	(21)	
	25-29 (13)	11	(85)	2	(15)	
	30-34 (10)	9	(90)	1	(10)	
	35-39 (8)	7	(88)	1	(12)	
	40-42 (2)	2	(100)	0	(0)	
Signs of sinus infection before surgery	No (74)	66	(89)	8	(11)	0.001
	Yes (26)	14	(54)	12	(46)	
Preoperative antibiotics	No (82)	67	(82)	15	(18)	0.254
	Yes (18)	13	(72)	5	(28)	
Drain insertion	No (59)	47	(80)	12	(20)	0.564
	Yes (41)	33	(81)	8	(19)	
Types of surgery for all patients	Primary closure (24)	20	(83)	4	(17)	0.265
	Karydakis closure (20)	16	(80)	4	(20)	
	Left opened (22)	18	(82)	4	(22)	
	Limberg (rotational flap) (34)	26	(76)	8	(24)	

All of our patients underwent one of four surgical techniques with 24 patients having sinus excision with primary midline closure, 22 patients having sinus excision with the wound left open, 20 patients having sinus excision with Karydakis operations, and 34 patients having sinus excision with Limberg flap closure. There was no significant difference between these four groups and the rate of recurrence (p-value = 0.26). However, the patients who underwent Limberg flap closure and had one or more of the signs of infection had a higher recurrence rate (77.8%) as compared with the other groups, on the other hand, patients who underwent Limberg flap closure and had no signs of infection had lower recurrence rate (8%) (Table 2).

**Table 2:** Correlation between different types of surgical procedures with the recurrence in two groups of patients with and without signs of sinus infection before surgery

Parameters	Recurrence of sinus			
	No, N: 80(%)		Yes, N: 20 (%)	
Type of surgery for all patients				
Primary closure (24)	20	(83)	4	(17)
Karydakis closure (20)	16	(80)	4	(20)
Left opened (22)	18	(82)	4	(22)
Limberg (rotational flap) (34)	26	(76)	8	(24)
Type of surgery for patients with signs of infection (26)				
Primary closure (4)	2	(50)	2	(50)
Karydakis closure (5)	4	(80)	1	(20)
Left opened (8)	5	(63)	3	(37)
Rhomboid (rotational flap) (9)	3	(33)	6	(67)
Type of surgery for patients with no signs of infection (74)				
Primary closure (20)	18	(90)	2	(10)
Karydakis closure (15)	12	(80)	3	(20)
Left opened (14)	13	(93)	1	(7)
Limberg (rotational flap) (25)	23	(92)	2	(8)

Forty-one patients had a drain inserted during the surgery which was removed within two days after operation. There was no significant difference as a result of putting a drain in the wound or not with the recurrence of the sinus (p-value 0.564) (Table 1).

## Discussion

Due to its high recurrence rate, which may reach as high as 30%, pilonidal sinus disease is considered a frustrating condition for many patients [7]. After the literature review, we noticed that there are variable results in the recurrence rate of the pilonidal sinus after excision, best described by Boshnaq et al. who reported a recurrence rate at 0%-7.4% in case series, and in comparative studies at 0%-8.3%, compared to 4%-37.7% for primary closure and 0%-11% for Karydakis flap surgery [8]. Some of the previous results demonstrated a low recurrence rate, such as a study conducted by Sevinç et al. which reported recurrence rates at 6% for both Limberg and Karydakis patients, and 4% for patients who underwent tension-free primary closure [9]. On the other hand, some studies reported a high recurrence rate such as Halleran et al. who found that the recurrence rates of pilonidal disease have reached as high as 30% [10]. Milone et al. concluded that the overall incidence of recurrence after sinus excision, which was followed by left open healing, midline closure, or out-midline closure, was 17.9%, 16.8%, and 10%, respectively [11]. Also, López et al. reported that nearly one of 10 patients who underwent pilonidal sinus excision developed recurrent disease requiring re-excision within two years post-surgery [12]. Another conclusion by Ekici et al. reported that recent studies have shown a rise in the recurrence rate, which started at 1% during the first-year follow-up and reached 14.3% during a two-year follow-up period [5]. This wide difference could be explained by Stauffer et al. who stated that the recurrence of the sinus after surgical excision is dependent on both the type of the surgical procedure performed and the follow-up time; both should be considered [6]. With our results, we can add that the presence of signs of sinus infection before surgery will also determine the possibility of recurrence. We reported a 20% recurrence rate among our patients over a three-year follow-up period post-surgery, which is matching with the upper limit of the results of the other studies. This could be explained by the fact that 26% of our patients had signs of sinus infection before surgery, which turned out to be a significant factor in the recurrence of the disease in our study.

The presence of signs of infection in the region of the pilonidal sinus before surgery in the form of purulent discharge, offensive odor, or a red and tender area, not including an abscess formation, was the only statistically significant finding in our study. It concludes a relative cause of the recurrence of the disease as nearly half of our patients who demonstrated signs of infection preoperatively (46%) had recurrence regardless of the type of surgical technique they underwent. To the best of our knowledge, the literature lacks previous studies that describe the relationship between the signs of infection present in the sinus region before surgery and its recurrence rate.

Previous research related to pilonidal sinus found that this disease is common in young males, which was also found in our study which showed a mean age of 24 years and a greater prevalence of the pilonidal sinus among males (81%). Our results showed that there is no statistical relationship between the age or the gender of the patients and the recurrence of the sinus after surgical excision, which follows almost all the previous studies, such as the one conducted by Almajid et al. who found that 93% of his pilonidal sinus patients were males and their mean age was 23±8 years [13]. Another study, by Ekici et al., found that among 303 patients with pilonidal sinus, 80.5% were male, and the mean age was 24 [5]. Also, Abo-Ryia et al. found in their study that males constituted 93.6% with a mean age of 22 years [14]. We had no patients admitted to our hospital with pilonidal sinus at this period of study who were over 42 years old.

In our retrospective study, we found no statistical relationship between the four types of surgical techniques, which were primary midline closure, Karydakis closure, Limberg flap, and left open method, and the recurrence of the sinus. Several previous studies were conducted to evaluate the differences between various surgical techniques about the recurrence of the sinus. Only a very few papers obtained statistical significance, such as Ekici et al. who found that in the primary closure method, the recurrence rate was statistically significant and higher than that of the other methods (p = 0.009) [5]. Also, Mahdy et al. stated that a significant difference between different surgical techniques after sinus excision with its recurrence was observed, flap reconstruction was superior to primary closure, and modified Limberg flap had better results [15]. The majority of other studies provided no statistical significance between the type of surgical technique and the recurrence similar to our finding. To illustrate this controversy in the results, we can compare a study by Al-Khamis et al., which concluded that no clear benefit has been shown for the lay open method over different surgical closure techniques, and a study by Rashidian et al. which considered the primary and Limberg flap closure superior to the lay open method in terms of recurrence and other complications [16,17]. On the other hand, most of the previous studies were in favor of the Limberg flap and Karydakis closure rather than the primary midline closure and the left-open method. This is well demonstrated in a study by Kanlioz et al. who recommended the Limberg flap technique as it had the lowest recurrence rate and was the most successful treatment method [18]. Another meta-analysis study by Bi et al. showed that both modified Limberg flap and off-midline closure had relatively low recurrence rates [4]. Similarly, Ray et al. stated that, although it failed to achieve statistical significance, Limberg flap closure seems to have a clinical advantage over both Karydakis and Bascom procedures in terms of reduced recurrence rate following excision of the sinus [19]. That was in contrast to Okuş et al. who found that the Limberg flap technique was as effective as a tension-free primary closure [20]. Also, Sevinç et al. had no statistical difference in their study, which showed a recurrence rate of 6% for Limberg and Karydakis procedures and 4% for tension-free primary closure procedures [9]. Another interesting study by Tocchi et al. showed that primary midline closure and wound drainage is an effective procedure in patients with uncomplicated sinuses, and flap techniques should be reserved for complicated cases only [21]. Similarly, Erkent et al. advised for primary midline closure which remains important for treatment, instead of observing that the recurrence rates due to inadequate excision are mostly observed in patients who underwent this technique [1]. Trying to find a conclusion to this debate, Enshaei et al. offered that each surgeon can decide the appropriate method of surgery according to the size of the sinus and the occupational status of the patients [3]. Similarly, Isik et al. concluded in their study that the preference of the surgeon and the patient’s request are effective factors as there is no gold standard approach has been determined to treat the disease [22].

Although there was no significant relationship in our study between the type of surgical technique and the recurrence, we had a relatively high rate of recurrent sinus infections in the group of patients who underwent Limberg flap closure (24%). That could be explained because most of the patients who underwent Limberg flap surgery and had signs of sinus infection before surgery had a higher rate of recurrence (67%) compared to the relatively low recurrence rate in the patients who underwent Limberg flap closure and had not shown signs of sinus infection before surgery (8%) (Table 2); thus, this has turned out to be a significant factor in the recurrence of the disease.

In terms of antibiotics and their significance in preventing the recurrence of the disease, we found that very few previous research studies were conducted evaluating the use of antibiotics in patients going for pilonidal sinus excision and its relation to the recurrence of the disease. Although Karip et al. in their study believed that antibiotic prophylaxis is necessary in patients scheduled for Karydakis flap repair, they did not achieve statistical significance [23]. Similarly, we found that the prescription of a course of oral antibiotics prior to hospital admission has no role in the recurrence of the sinus after excision, and there was also no statistical significance found.

Regarding drain insertion into the wound after sinus excision, in our study, we did not find a statistical relationship between the insertion of a drain and the recurrence of the disease. Similar to our results, Mentes et al. concluded that the use of a closed suction drain in patients who underwent Limberg flap operation is not necessary and will not affect the recurrence rate [24]. However, another study by Brusciano et al. reported that suction drain insertion and skin closure in patients with sacrococcygeal pilonidal sinus offers an effective healing rate as well as low recurrence [25].

Our study is a retrospective one which is considered one of our limitations. This was because we had to collect significant data from more than 100 patients over three years. Another limitation of our study was that the management of these patients was carried out by different surgeons over three years. On the other hand, we had a follow-up period of three years post-surgery which provided us with more broad data about the rate of the recurrence.

## Conclusions

The presence of any of the signs of sinus infection before surgery was the only statistically significant predictor for the recurrence of the disease regardless of the surgical technique used. On the other hand, there is no correlation between the type of surgical technique, the preoperative course of antibiotics, and the use of a drain after sinus excision with the recurrence of the pilonidal sinus.
